# Nutrient Loads Flowing into Coastal Waters from the Main Rivers of China (2006–2012)

**DOI:** 10.1038/srep16678

**Published:** 2015-11-19

**Authors:** Yindong Tong, Yue Zhao, Gengchong Zhen, Jie Chi, Xianhua Liu, Yiren Lu, Xuejun Wang, Ruihua Yao, Junyue Chen, Wei Zhang

**Affiliations:** 1 School of Environmental Science and Engineering, Tianjin University, Tianjin, 300072, China; 2 Ministry of Education Laboratory for Earth Surface Processes, Peking University, Beijing, 100871, China; 3 Chinese Academy for Environmental Planning, Beijing, 100012, China; 4 School of Environment and Natural Resources, Renmin University of China, Beijing, 100872, China; 5 College of Urban and Environmental Sciences, Peking University, Beijing, 100871, China; 6 Yang Ming Institute, Ningbo University, Ningbo, 315122, China

## Abstract

Based on monthly monitoring data of unfiltered water, the nutrient discharges of the eight main rivers flowing into the coastal waters of China were calculated from 2006 to 2012. In 2012, the total load of NH_3_-N (calculated in nitrogen), total nitrogen (TN, calculated in nitrogen) and total phosphorus (TP, calculated in phosphorus) was 5.1 × 10^5^, 3.1 × 10^6^ and 2.8 × 10^5^ tons, respectively, while in 2006, the nutrient load was 7.4 × 10^5^, 2.2 × 10^6^ and 1.6 × 10^5^ tons, respectively. The nutrient loading from the eight major rivers into the coastal waters peaked in summer and autumn, probably due to the large water discharge in the wet season. The Yangtze River was the largest riverine nutrient source for the coastal waters, contributing 48% of the NH_3_-N discharges, 66% of the TN discharges and 84% of the TP discharges of the eight major rivers in 2012. The East China Sea received the majority of the nutrient discharges, i.e. 50% of NH_3_-N (2.7 × 10^5^ tons), 70% of TN (2.2 × 10^6^ tons) and 87% of TP (2.5 × 10^5^ tons) in 2012. The riverine discharge of TN into the Yellow Sea and Bohai Sea was lower than that from the direct atmospheric deposition, while for the East China Sea, the riverine TN input was larger.

“Dead zones” in coastal areas have spread exponentially since the 1960s and have caused serious consequences for ecosystem functioning^1^. One important cause for the dead zones is coastal eutrophication^1^,^2^,^3^. Due to population growth, rapid industrialization and urbanization, numerous previously pristine, unimpacted coastal waters have undergone a transformation to more mesotrophic and eutrophic conditions. At present, eutrophication offshore has become a global concern, and also one of the most prominent environmental problems in China^2^,^3^. In 2012, the area affected by eutrophication was estimated to be 9.8 × 10^4^ km^2^ in China, increasing by 2.4 × 10^4^ km^2^ from 2011^4^. The areas with the most severe eutrophication always occurred near the estuaries of the main rivers^4^. Coastal eutrophication could be attributed to the enrichment of nutrients in the water, such as nitrogen and phosphorus^5^,^6^, and the nutrient enrichment could increase the productivity of phytoplankton, ultimately leading to harmful algal blooms (HABs)^7^,^8^. It has been reported that red tides occurred 73 times offshore from China, and the coastal area affected by red tides could be as high as 7971 km^2^ in 2012^4^.

The sources of nutrients flowing into the seas could be generally divided into non-point sources (such as agriculture diffuses) and point sources (such as industrial and sewage sources)^2^. In order to feed the growing population in China, more grains need to be planted. The grain production in 2005 was 70% higher than in the 1980s^9^. However, it has been estimated only 20–35% of the nitrogenous fertilizer used could be assimilated by the crops, while the majority was discharged into the environment^10^. Moreover, as a result of improving living standards in China, many people have shifted their dietary preferences toward animal-derived products^11^,^12^. These dietary changes were also associated with increased nutrient inputs for agriculture^13^,^14^.

Wastewater and other industrial emissions caused by the urbanization and industrialization also increased nutrient inputs to the rivers and coastal waters. From 2006 to 2012, the wastewater produced in China increased from 536.8 × 10^8^ to 684.6 × 10^8^ tons^15^,^16^. Compared with the rapid growth in wastewater amounts, wastewater treatment facilities could not keep up with the urbanization progress. From 2000 to 2005, only 30–45% of the emitted wastewater was treated before being discharged into waters^17^. After 2006, in order to control the pollution of point sources, the Chinese government began to emphasize the construction of wastewater treatment plants. The number of wastewater treatment plants increased from 1040 in 2006, to 2496 in 2010^18^, with the daily sewage treatment capacity reaching 1.25 × 10^8^ m^3^ in 2010. However, the growing number of wastewater treatment facilities still can’t meet the demands of increasing wastewater. In 2010, the percentages of treated sewage in urban and rural in China were only about 82% and 60%, respectively^18^.

The nutrients emitted from both point and non-point sources are ultimately discharged to the coastal water through the rivers. Currently, most statistical studies for Chinese cases only focus on changes in nutrients concentrations or transport in small-scale watersheds^19^,^20^,^21^,^22^. For instance, Chen *et al.* (2000) found seasonal variances of nitrogen content varied with watersheds in the Yangtze River system, and the difference of nitrogen contamination level was related to the regional population and economic development^23^. Li *et al.* (2014) concluded that increased nutrient loads from the Yangtze River had led to increased Harmful Algal Blooms^3^.

The large scale nutrient export estimation to the coastal water always relied on model estimation. For example, based on the NEWS model, Qu and Kroeze (2010) calculated the export of dissolved nitrogen and phosphorus of the rivers in China, and their results suggested between 1970 and 2000 the dissolved nitrogen and phosphorus export to the coastal water increased significantly, while the export of other nutrients changed less^24^. Strokal *et al.* (2014) modeled the riverine inputs of nitrogen and phosphorus, and suggested dissolved nitrogen and phosphorus inputs to the Bohai Gulf, Yellow Sea and South China Sea increased by a factor of 2–5 between 1970 and 2000^14^. However, although the modeling research has been carried out recently, the knowledge about the riverine nutrient discharges into the seas in China is still limited.

In this study, monthly monitoring data (unfiltered water) of eight major Chinese rivers was used to estimate the nutrient flux into the seas of China from 2006 to 2012. The nutrients that were measured in include NH_3_-N (calculated in nitrogen), total nitrogen (TN, calculated in nitrogen) and total phosphorus (TP, calculated in phosphorus). The temporal and spatial variations of the nutrient concentrations of the eight rivers were analyzed. The monthly, seasonal and annual nutrient discharges into the coastal waters of China from 2006 to 2012 were calculated, and contributions of nutrient discharges from different rivers were compared. The impacts of construction on wastewater treatment facilities were also discussed.

## Results

### Concentrations of NH_3_-N, TN and TP

**Figure 1:**
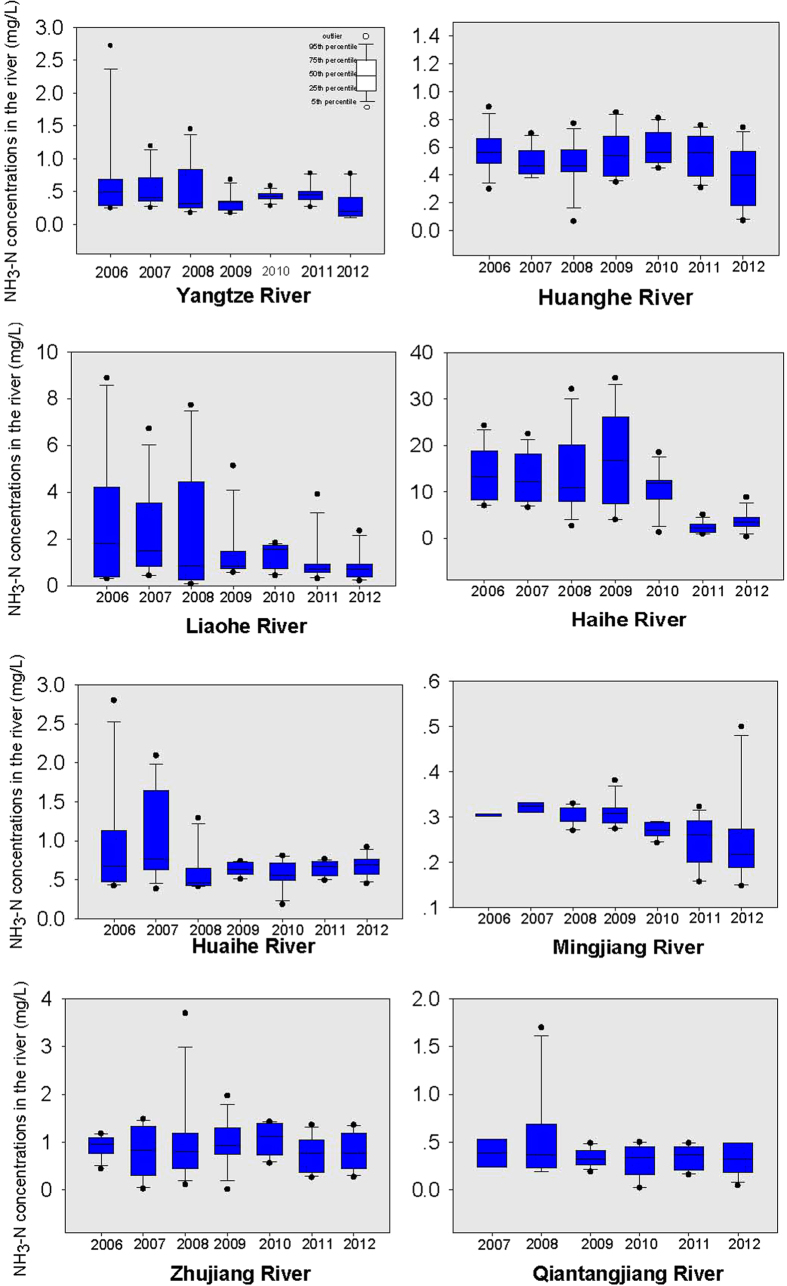
NH3-N concentrations in the rivers from 2006 to 2012.

The yearly NH_3_-N concentrations in the eight large rivers are presented in Fig. 1. In 2012, the highest NH_3_-N concentration was found in the Haihe River, with an average of 3.7 mg/L, and the lowest was found in the Minjiang River, with an average of 0.3 mg/L. Generally, the NH_3_-N concentrations have decreased since 2006. For example, the average NH_3_-N concentration for the Haihe River in 2006 was 14.0 mg/L (the monthly values ranging from 7.0 to 24.3 mg/L), decreasing to 3.7 mg/L (ranging from 0.3 to 8.8 mg/L) in 2012. For the Liaohe River, the average NH_3_-N concentration was 3.7 mg/L (ranging from 0.4 to 9.0 mg/L) in 2006, decreasing to 1.0 mg/L (ranging from 0.2 to 2.4 mg/L) in 2012. For the Yangtze River, the average concentrations were 0.7 mg/L (ranging from 0.2 to 2.7 mg/L) in 2006 and decreased to 0.3 mg/L in 2012. Compared with the rivers in North China, the NH_3_-N concentrations were generally lower for the rivers in South China. In 2012, the average monthly NH_3_-N concentrations of the four rivers in northern China (Liaohe, Huanghe, Haihe and Huaihe Rivers) were significantly higher than those of the rivers in the south (Yangtze, Qiantangjiang, Minjiang and Zhujiang Rivers).

**Figure 2:**
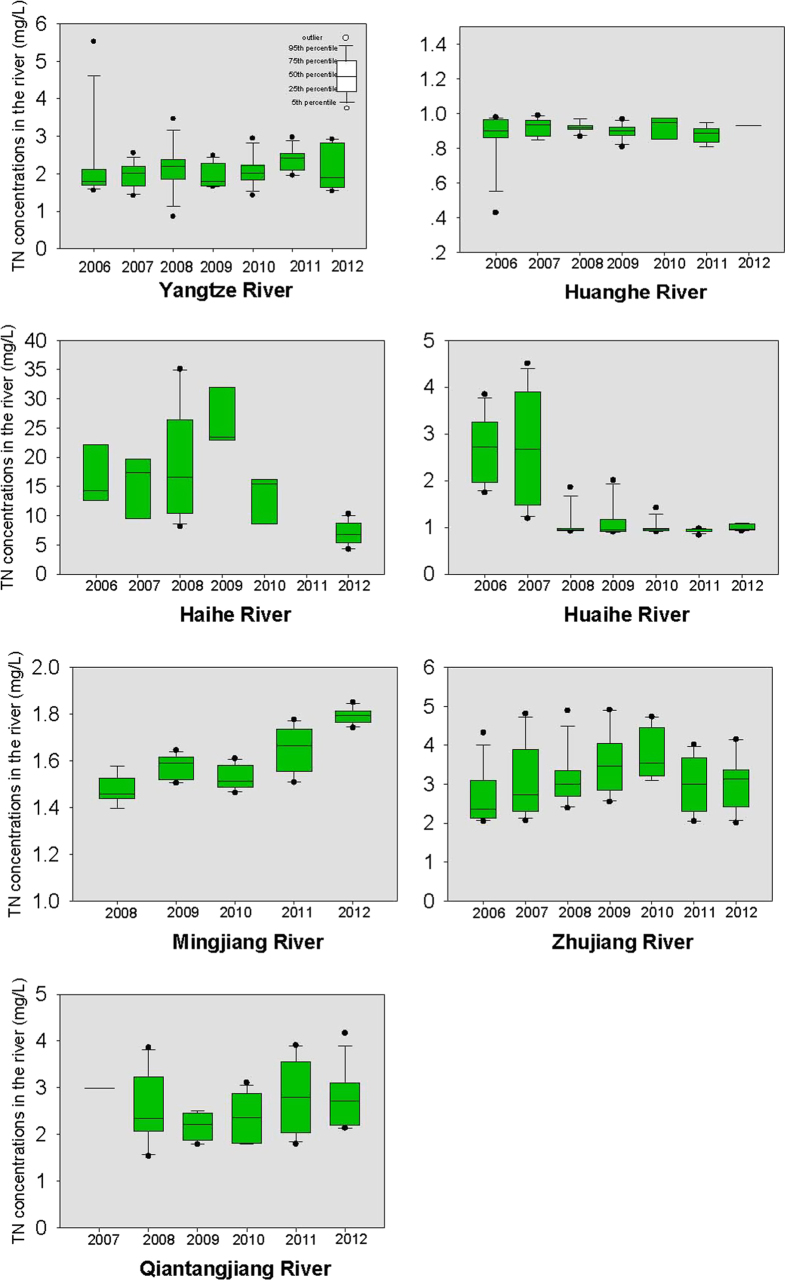
TN concentrations in the rivers from 2006 to 2012.

The yearly TN concentrations in the seven large rivers (excluding the Liaohe River) are provided in Fig. 2. During the period between 2006 and 2012, the TN concentrations were generally lower than 5 mg/L for a majority of the rivers (except the Haihe River). In 2012, the highest TN concentration was found in the Haihe River, with an average of 7.0 mg/L, and the lowest concentration was found in the Huanghe River, with an average of 0.9 mg/L. The TN concentrations showed a decreasing trend from 2006 to 2012. For the Haihe River, the average TN concentration was 17.0 mg/L (ranging from 11.7 to 28.8 mg/L) in 2006, and decreased to 7.0 mg/L (ranging from 4.3 to 10.3 mg/L) in 2012. For the Huaihe River, a remarkable decreasing trend was observed from 2007 to 2008. The average TN concentration in the Huaihe River in 2007 was 2.7 mg/L (ranging from 1.2 to 4.5 mg/L), and decreased to 1.0 mg/L (ranging from 0.9 to 1.8 mg/L) in 2008. For the Huanghe River, TN concentrations were quite stable from 2006 to 2012, staying near 1.0 mg/L.

**Figure 3:**
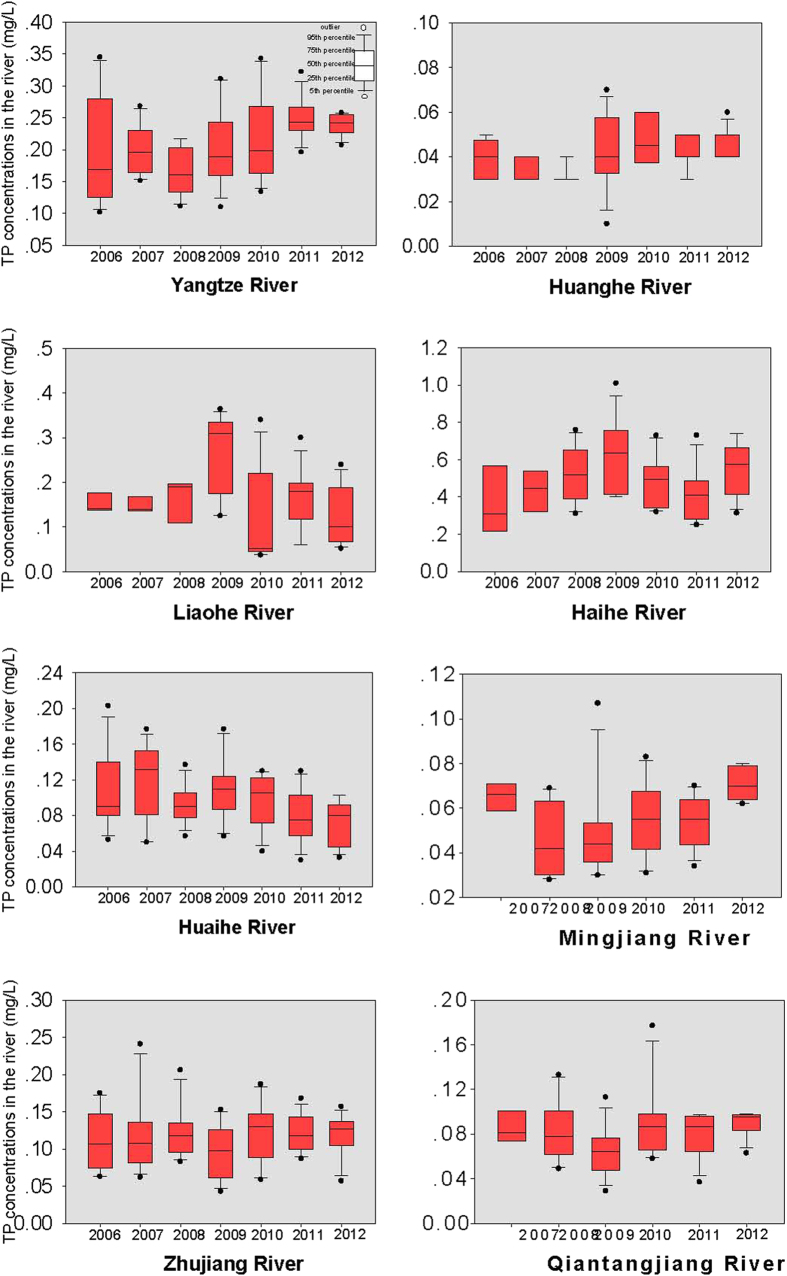
TP concentrations in the rivers from 2006 to 2012.

The yearly TP concentrations in the eight large rivers are provided in Fig. 3. In 2012, the highest TP concentration was observed in the Haihe River, with an average concentration of 0.6 mg/L (ranging from 0.3 to 0.7 mg/L), and the lowest TP concentration occurred in the Huanghe River, with an average concentration of 0.04 mg/L (ranging from 0.04 to 0.06 mg/L). For the Huaihe River, the average TP concentration decreased from 0.11 mg/L (ranging from 0.07 to 0.20 mg/L) in 2006 to 0.07 mg/L (ranging from 0.03 to 0.10 mg/L) in 2012. However, for the Minjiang River, similar to TN, a slight increase in TP concentration was found after 2008. In 2008, the average TP concentration was 0.05 mg/L (ranging from 0.03 to 0.07 mg/L), and increased to 0.07 mg/L (ranging from 0.06 to 0.08 mg/L) in 2012.

### Nutrient loads

The yearly loads of NH_3_-N, TN and TP of the selected rivers flowing into the coastal waters of China from 2006 to 2012 were calculated and are presented in Table 1. In 2012, the total load of NH_3_-N, TN and TP of the selected rivers was 5.1 × 10^5^, 3.1 × 10^6^ and 2.8 × 10^5^ tons, respectively. In 2006, the nutrient load was 7.4 × 10^5^, 2.2 × 10^6^ and 1.6 × 10^5^ tons for NH_3_-N, TN and TP, respectively. The Yangtze River was the largest riverine nutrient source for the coastal waters. In 2012, Yangtze River contributed 48% of the NH_3_-N discharges, 66% of the TN discharges and 84% of the TP discharges of the eight major rivers. The Zhujiang River was the second greatest nutrient source for the coastal waters. In 2012, Zhujiang River contributed about 43% of the NH_3_-N discharges, 26% of the TN discharges and 12% of the TP discharges. The Huanghe River is the second longest river in China, but the nutrient discharge to coastal waters was much lower than that of the Yangtze and Zhujiang Rivers. The small nutrient discharge could possibly be attributed the low water discharge of the Huanghe River, which was caused by excessive water use and climate change in the Huanghe River Basin^25^,^26^.

The nutrient loading varied a lot among different seas (Table 2). The East China Sea received the majority of the nutrient discharges. In 2012, about 50% of the NH_3_-N (2.7 × 10^5^ tons), 70% of the TN (2.2 × 10^6^ tons), 87% of the TP (2.5 × 10^5^ tons) loads were discharged into the East China Sea, while only 3% of the NH_3_-N loads and less than 1% of TN and TP loads flowed into the Bohai Sea (Table 2). The South China Sea is also an important destination for nutrients. In 2012, about 2.2 × 10^5^ tons of NH_3_-N, 8.0 × 10^5^ tons of TN and 3.5 × 10^5^ tons of TP were discharged into the South China Sea.

## Discussion

**Figure 4:**
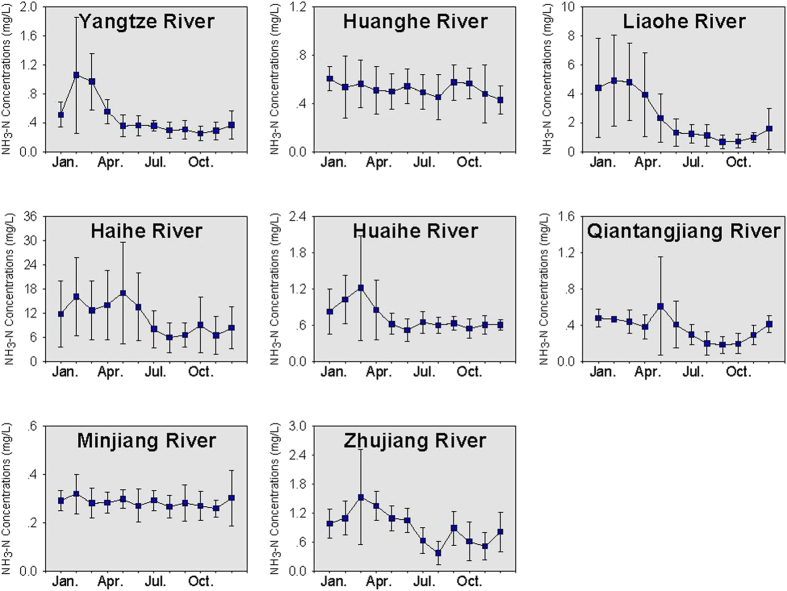
Monthly variation of NH3-N concentrations in the rivers. * The error bar refers to the standard deviation of the nutrient concentrations from 2006 to 2012.

**Figure 5:**
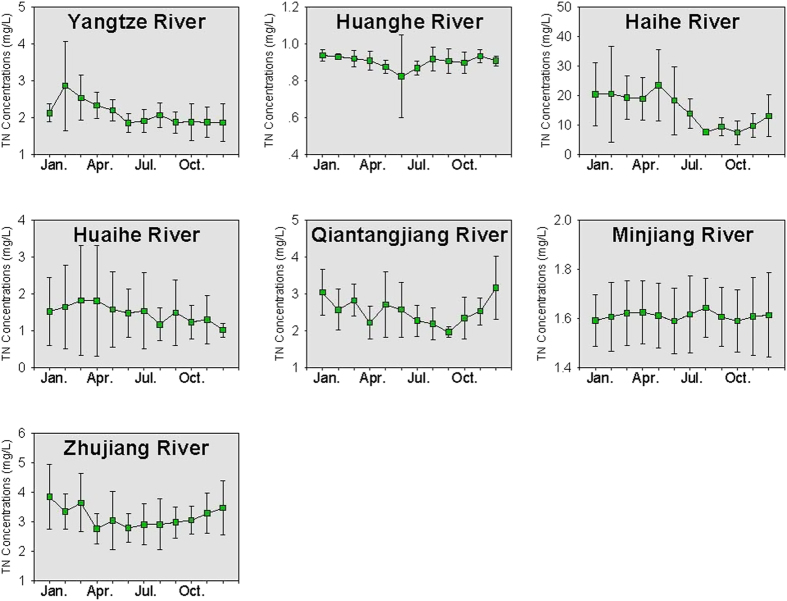
Monthly variation of TN concentrations in the rivers. * Liaohe River is not included due to lack of data. * The error bar refers to the standard deviation of the nutrient concentrations from 2006 to 2012.

**Figure 6:**
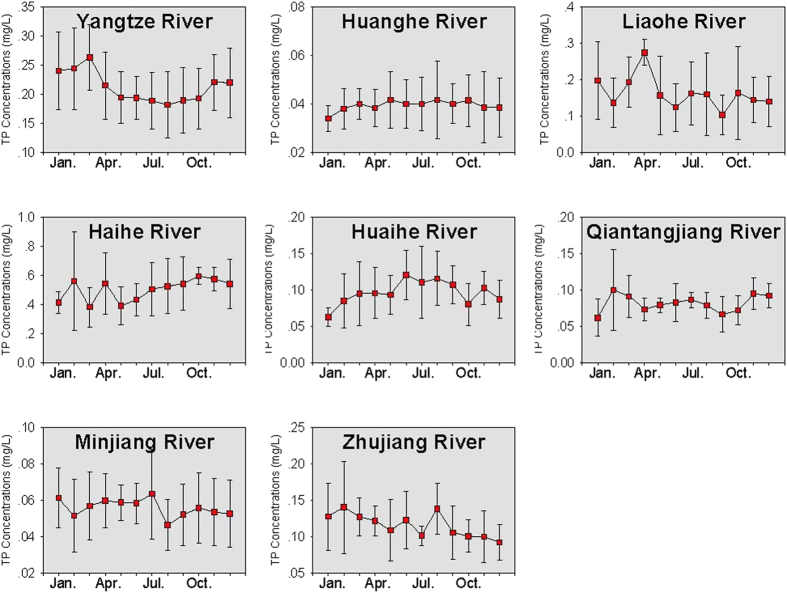
Monthly variation of TP concentrations in the rivers. *The error bar refers to the standard deviation of the nutrient concentrations from 2006 to 2012.

The monthly variation of NH_3_-N, TN and TP concentrations in the eight rivers is provided in Figs 4, 5 and 6, respectively. For the Yangtze River, Liaohe River and Huaihe River, an annual peak in NH_3_-N concentration was observed in February–March (dry season), indicating high NH_3_-N concentrations at low flows. For these rivers, a negative relationship was found between the monthly water discharge and NH_3_-N concentrations (p = 0.002). As suggested by previous studies, the negative relationship could indicate the effects of point sources (such as discharge of sewage and industrial wastewater) on the NH_3_-N concentrations in the rivers^27^. For the Huanghe River and Minjiang River, the correlation between NH_3_-N and water discharge was not significant, and NH_3_-N concentrations showed small variations between months, implying that these rivers could have a mixture of diffuse sources, point sources and non-point sources^23^.

Monthly variation of TN concentrations in the rivers is shown in Fig. 5. For the Yangtze River, a monthly variation similar to NH_3_-N was found, and the annual peak always occurred in February-March (dry season). For the Qiantangjiang and Zhujiang Rivers, a “double-peak” of TN concentrations was observed. The peak value of TN concentrations appeared in the autumn and winter (or in the early spring), and the lowest value was usually detected in the summer. For example, in the Zhujiang River in 2007, a double-peak occurred in August and December, with a value of 4.81 and 4.53 mg/L, respectively, while the lowest concentration occurred in April, with a value of 2.07 mg/L. This phenomenon was similar to that reported in a previous study^3^, and has been attributed to the intense mixing in the water masses^3^,^28^. Generally, nutrient concentrations in bottom water were fairly abundant due to the remineralization of organic matter. The vertical mixing from surface to bottom water was stronger in autumn and winter than in spring and summer. Hence, nutrient concentrations peaked during autumn and winter.

In all the dissolved inorganic nitrogen (DIN) in the rivers, the nitrate is the dominant form, usually occupying for over 80%^29^,^30^, while in this study, a high proportion of NH_3_-N in TN was observed. The difference of nutrient concentrations between their studies and ours could possibly be attributed to the difference of water pretreatment. In the studies by Gao *et al.* and Liu *et al.*^29^,^30^, filtered water samples were used for the nutrient analysis, while in this study, the unfiltered water samples were analyzed for the NH_3_-N, TN and TP. Nutrients in the suspended particles have proved to account for a large proportion of nutrients in the water. It was reported that the particulate phosphorus (PP) occupied for 62–99% of the total phosphorus (TP) in the Huanghe River^31^. TN concentrations in the rivers were also much higher than DIN. In Yangtze River, DIN concentrations in the surface water were 0.53 mg/L, while the corresponding TN concentrations were 1.15 mg/L^32^. Compared with the dissolved nitrogen species such as NO_3_-N, the suspended particles in the water could adsorb the NH_3_-N much more easily^33^,^34^, leading to a higher proportion of NH_3_-N in the TN.

For the Yangtze, Huaihe, Qiantangjiang and Minjiang Rivers, a positive relationship was found between the TN and TP concentrations (p = 0.001, 0.02, 0.01, 0.003, respectively). This indicated that the TN and TP concentrations were affected by similar factors. However, for the Zhujiang River, no significant correlation was observed between the TP and TN concentrations (p = 0.56).

The yearly loading of NH_3_-N into coastal waters showed a decreasing trend from 2006 to 2012 (Table 1). Compared with 2006, the riverine NH_3_-N loads decreased by 31% in 2012, from 7.4 × 10^5^ to 5.1 × 10^5^ tons. As the largest NH_3_-N contributor to the coastal seas, the Yangtze River was showed a decrease in NH_3_-N discharge from 4.4 × 10^5^ tons in 2006 to 2.4 × 10^5^ tons in 2012. The decreasing trend of NH_3_-N loads was consistent with the decrease of NH_3_-N concentrations in the river. The decline of NH_3_-N discharges could possibly be attributed to the fact that more and more sewage treatment plants have been put into operation in China. According to a report from the Ministry of Housing and Urban-Rural Development, the sewage treatment capacity of cities increased from 6.5 × 10^7^ m^3^/day in 2006 to 1.0 × 10^8^ m^3^/day in 2010^18^. The reduction of NH_3_-N is a priority in sewage treatment plants, and the NH_3_-N abatement by the sewage treatment plants increased from 2.6 × 10^5^ tons in 2006 to 7.7 × 10^5^ tons in 2010^18^. In China, the total anthropogenic emission of NH_3_-N (including industrial source, domestic source, agricultural source, etc.) in 2011 and 2012 was estimated to be 2.6 × 10^6^ and 2.5 × 10^6^ tons, respectively^16^,^35^. According to our calculation, the riverine NH_3_-N discharge in 2011 and 2012 was 4.7 × 10^5^ and 4.9 × 10^5^ tons, respectively. This indicates that approximately 18% and 19% of the anthropogenic NH_3_-N emission was released into the coastal waters through rivers. The direct NH_3_-N discharges into the coastal waters from sewage treatment plants in coastal cities were reported to be 2.3 × 10^4^ tons in 2010, 1.6 × 10^4^ tons in 2011, and 1.2 × 10^4^ tons in 2012, respectively^16^,^35^,^36^. Compared with the riverine NH_3_-N discharge, the direct NH_3_-N discharge from the coastal cities was minor.

For TN and TP discharges, an increase was found in 2012 compared to 2006 (Table 1). In 2006, the TN and TP discharges from the eight major rivers into the coastal waters were 2.2 × 10^6^ and 1.6  × 10^5^ tons, respectively, and increased to 3.1 × 10^6^ and 2.8 × 10^5^ tons in 2012. For the Yangtze River, the discharge of TN increased from 1.4 × 10^6^ tons in 2006 to 2.0 × 10^6^ tons in 2012, and the TP discharge increased from 1.2 × 10^5^ in 2006 to 2.4 × 10^5^ tons in 2012. It was reported that the average annual loading of TN from Mississippi-Atchafalaya River into Mexico Gulf was 1.6 × 10^6^ tons^37^. In this study, the TN loading from the Yangtze River was approaching that of the Mississippi River. The direct TP discharge from the sewage plants along the coast into the coastal waters was reported to be 2901 tons in 2010, 2447 tons in 2011 and 2196 tons in 2012^16^,^35^,^36^. Compared with the riverine TP discharge, the direct TP discharge from sewage plants of the coastal cities into the coastal waters was trivial.

The nutrient loading from the eight major rivers into the coastal waters always peaked in summer and autumn (Table S1 in the Supplementary Information). For the Yangtze River, from 2006 to 2012, the average TN load was 5.0 × 10^5^ tons (ranging from 3.7 × 10^5^ to 6.8 × 10^5^ tons) in summer and 6.2 × 10^5^ tons (ranging from 3.6 × 10^5^ to 7.9 × 10^5^ tons) in autumn, higher than the TN load in spring (averaging 9.8 × 10^4^ tons, ranging from 6.3 × 10^4^ to 1.8 × 10^5^ tons). The TN and TP loads in summer and autumn accounted for 64% and 67% of the annual discharge, respectively. For the Zhujiang River, the average TN load was 2.9 × 10^5^ tons (ranging from 1.9 × 10^5^ to 4.3 × 10^5^ tons) in summer and 2.7 × 10^5^ tons (ranging from 1.0 × 10^5^ to 3.7 × 10^5^ tons) in autumn, higher than the TN load in spring (averaging 1.0 × 10^5^ tons, ranging from 0.7 × 10^5^ to 1.4 × 10^5^ tons). The monthly loads of NH_3_-N, TN and TP of the selected rivers have the same trend of incremental value from dry season to wet season (Figure S1 in the Supplementary Information). For instance, for the Yangtze River and Zhujiang River, a significant relationship between the water discharge and nutrient loads was observed (p = 0.003). Hence, the high nutrient loads in the wet season could possibly be attributed to the large water discharges.

Previous studies reported that atmospheric nitrogen deposition played an important role in the nitrogen cycles in coastal ecosystems in China^38^. Bashkin *et al.* (2002) estimated that the atmospheric deposition of inorganic nitrogen to the Yellow Sea and Bohai Sea was 1.06 × 10^6^ tons/year^39^. In our calculation, the riverine discharge of total nitrogen into the Yellow Sea and Bohai Sea was 3.7 × 10^4^ tons, much lower than those from direct atmospheric deposition. Previous studies reported that atmospheric deposition of dissolved inorganic nitrogen to the East China Sea was 1.75 × 10^6^ tons/year^40^, and this value is 0.78 times the riverine total nitrogen inputs (2.2 × 10^6^ tons) in 2012. These results indicated that direct atmospheric deposition was the dominant source of nitrogen in the Yellow Sea and Bohai Sea, while for the East China Sea, the riverine discharges had higher contributions compared with atmospheric deposition.

## Methods

### Study area

**Figure 7:**
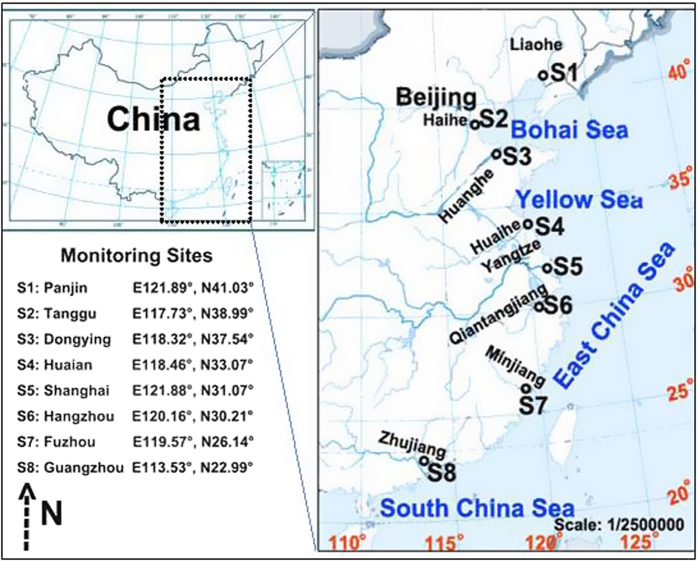
Locations of the sampling sites and study area (ArcGIS, version 10.2).

In this study we examined the eight main rivers flowing into the coastal waters of China include the Yangtze River, Huanghe River, Liaohe River, Haihe River, Huaihe River, Qiangtangjiang River, Minjiang River and Zhujiang River (Table 3 and Fig. 7). The total drainage area of the eight rivers was 319.6 × 10^4^ km^2^ (Table 3), occupying one third of the land area of China^41^. The eight rivers accounted for the majority of water discharge into the coastal waters in China. The annual water discharge of the rivers decreases from the south to the north. For example, the annual water discharge of Zhujiang River was 2833 × 10^8^ m^3^, 182 times as much as the water discharge of the Haihe River (Table 3). The peak of water discharge in the selected rivers occurred in the period from April to September (wet season)^41^. The water discharge information of the rivers is presented in the Supplementary Information (Figure S2).

Due to the “west high and east low” terrains, the rivers mostly flow from the west to the east. Throughout the length of their courses, the rivers receive pollutants from point and non-point sources, and discharge into coastal waters. The rivers discharge into different seas, including the Bohai Sea, Yellow Sea, East China Sea and South China Sea, and these seas cover a total area of about 4.7 million km^2^ 38. The East China Sea received 3/4 of the total water discharge.

### Data source and calculation

Ammonia nitrogen (NH_3_-N), total nitrogen (TN) and total phosphorus (TP) were monitored and collected every month from 2006 to 2012 by the China Ministry of Environmental Pollution, and the monitoring stations were located at the estuaries of the eight main rivers (shown in Fig. 7). The field sampling was carried out according to “Technical Specifications Requirements for Monitoring of Surface Water and Waste Water, in China (HJ/T 91-2002)” 42. In brief, for the rivers with a width less than 50 m, a vertical sampling was set at the middle thread of channel. For the rivers with a width between 50 and 100 m, two vertical sampling were set at both left and right sides (at the place with appreciable currents), respectively. For the rivers with a width more than 100 m, three vertical sampling were set at the left side, middle thread of channel and right side, respectively. In order to avoid the impacts of point sources, the sampling was carried out at the site which could represent the general nutrient conditions, but away from the point sources such as sewage outfalls. Specifically, water mixture samples (surface: 50 cm under the surface; middle: 1/2 of the river depth; bottom: 50 cm above the riverbed) were collected using a Polyethylene bailer for each site at the ebb time, when the river is naturally flowing. The sampling equipment was cleaned thoroughly with deionized water between each site to avoid cross contamination. About 0.5~1 L water samples were collected each time. As soon as the waters samples were collected, H_2_SO_4_ (GR) was added into the samples to make pH in the samples lower than 2 to avoid the impacts form the microbial processes, etc. The water samples were kept in the refrigerator before analysis (at the temperature of 4 °C). The laboratory measurement was carried out according to the “Environmental Quality Standard for Surface Water, China (GB 3838-2002)” 43. For the riverine nutrient discharges, besides the dissolved nutrients, nutrients in the suspended particles should also be considered. Hence, in this study, the unfiltered water samples were used for analysis. The measurement was carried out within 24 hours for NH_3_-N and TP, and 7 days for TN. The limits of detection for NH_3_-N, TP and TN in the water samples were 0.01, 0.01 and 0.05 mg/L, respectively.

The annual nutrient discharges were calculated by the monthly nutrient concentrations and water discharge. The following formula (E.g. 1) was used to estimate the annual nutrient fluxes (F_Nutrient_ (tons)) from 2006 to 2012. C_Nutrient_ (mg/L) and V_Runoff_ (10^8^ m^3^) refer to the monthly concentrations of nutrients and water discharge in the selected rivers, respectively. 100 (tons·L/(mg·m^3^)) is the unit conversion factor. The water discharge information was collected at the hydrological stations of the river estuary, and these sites were also near the water monitoring sites in our study.


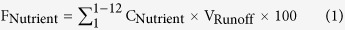


## Additional Information

**How to cite this article**: Tong, Y. *et al.* Nutrient Loads Flowing into Coastal Waters from the Main Rivers of China (2006-2012). *Sci. Rep.* **5**, 16678; doi: 10.1038/srep16678 (2015).

## Supplementary Material

Supplementary Information

## Figures and Tables

**Table 1:** Yearly nutrient loads of NH_3_-N, TN and TP into coastal seas during 2006–2012 (tons).

	Yangtze	Huanghe	Liaohe	Haihe	Huaihe	Qiantangjiang	Minjiang	Zhujiang	Total
NH_3_-N									
2006	4.37E + 05	1.19E + 04	4.48E + 03	2.05E + 00	2.50E + 04	—*	1.72E + 04	2.44E + 05	7.40E + 05
2007	3.66E + 05	9.36E + 03	2.25E + 03	2.66E + 00	3.51E + 04	4.89E + 03	1.05E + 04	2.13E + 05	6.42E + 05
2008	3.48E + 05	6.71E + 03	3.96E + 03	3.77E + 00	1.48E + 04	1.02E + 04	1.36E + 04	2.89E + 05	6.86E + 05
2009	2.56E + 05	7.18E + 03	1.71E + 03	6.79E + 00	8.45E + 03	4.87E + 03	1.17E + 04	2.00E + 05	4.89E + 05
2010	4.28E + 05	1.07E + 04	9.75E + 03	2.86E + 00	1.88E + 04	1.03E + 04	2.37E + 04	2.88E + 05	7.89E + 05
2011	2.98E + 05	1.02E + 04	2.52E + 03	1.16E + 00	6.17E + 03	3.96E + 03	7.79E + 03	1.39E + 05	4.68E + 05
2012	2.44E + 05	1.25E + 04	2.03E + 03	2.23E + 00	7.28E + 03	9.63E + 03	1.64E + 04	2.19E + 05	5.11E + 05
TN									
2006	1.35E + 06	1.61E + 04	—	2.47E + 03	7.14E + 04	—	—	7.74E + 05	2.22E + 06
2007	1.53E + 06	1.81E + 04	—	3.06E + 03	1.02E + 05	2.33E + 04	—	7.05E + 05	2.38E + 06
2008	1.72E + 06	1.36E + 04	—	5.80E + 03	2.90E + 04	4.18E + 04	7.04E + 04	1.02E + 06	2.90E + 06
2009	1.49E + 06	1.21E + 04	—	7.88E + 03	1.41E + 04	3.17E + 04	5.83E + 04	7.52E + 05	2.37E + 06
2010	2.09E + 06	1.37E + 04	—	2.67E + 03	3.12E + 04	6.59E + 04	1.34E + 05	9.52E + 05	3.29E + 06
2011	1.61E + 06	2.07E + 04	—	—	9.22E + 03	4.58E + 04	4.94E + 04	4.96E + 05	2.24E + 06
2012	2.04E + 06	2.15E + 04	—	4.72E + 03	1.08E + 04	7.52E + 04	1.23E + 05	8.02E + 05	3.08E + 06
TP									
2006	1.22E + 05	7.91E + 02	2.29E + 02	4.73E + 01	3.22E + 03	—	—	3.34E + 04	1.60E + 05
2007	1.56E + 05	6.64E + 02	1.54E + 02	1.06E + 02	5.78E + 03	1.13E + 03	2.08E + 03	2.52E + 04	1.91E + 05
2008	1.43E + 05	4.59E + 02	5.32E + 02	1.98E + 02	2.67E + 03	1.34E + 03	2.06E + 03	4.27E + 04	1.93E + 05
2009	1.48E + 05	6.08E + 02	3.51E + 02	2.56E + 02	1.62E + 03	9.51E + 02	2.25E + 03	2.00E + 04	1.74E + 05
2010	2.02E + 05	8.69E + 02	4.72E + 02	1.45E + 02	3.34E + 03	2.75E + 03	5.61E + 03	3.67E + 04	2.52E + 05
2011	1.67E + 05	1.01E + 03	4.00E + 02	2.09E + 02	8.29E + 02	1.30E + 03	1.71E + 03	2.17E + 04	1.94E + 05
2012	2.38E + 05	1.26E + 03	2.78E + 02	4.24E + 02	9.07E + 02	2.60E + 03	5.34E + 03	3.47E + 04	2.83E + 05

^*^Data was not monitored.

**Table 2:** Nutrient loads from the larger rivers into coastal seas during 2006–2012 (tons).

		East China Sea	Bohai Sea	Yellow Sea	South China Sea
2012	NH_3_-N	2.70E + 05	1.70E + 04	7.30E + 03	2.20E + 05
	TN	2.20E + 06	2.60E + 04	1.10E + 04	8.00E + 05
	TP	2.50E + 05	2.00E + 03	9.10E + 02	3.50E + 04
2011	NH_3_-N	3.10E + 05	1.38E + 04	6.17E + 03	1.39E + 05
	TN	1.71E + 06	2.07E + 04	9.22E + 03	4.96E + 05
	TP	1.70E + 05	1.62E + 03	8.29E + 02	2.17E + 04
2010	NH_3_-N	4.62E + 05	2.33E + 04	1.88E + 04	2.88E + 05
	TN	2.29E + 06	1.64E + 04	3.12E + 04	9.52E + 05
	TP	2.11E + 05	1.49E + 03	3.34E + 03	3.67E + 04
2009	NH_3_-N	2.72E + 05	1.57E + 04	8.45E + 03	2.00E + 05
	TN	1.58E + 06	1.99E + 04	1.41E + 04	7.52E + 05
	TP	1.51E + 05	1.22E + 03	1.62E + 03	2.00E + 04
2008	NH_3_-N	3.71E + 05	1.44E + 04	1.48E + 04	2.89E + 05
	TN	1.83E + 06	1.94E + 04	2.90E + 04	1.02E + 06
	TP	1.47E + 05	1.19E + 03	2.67E + 03	4.27E + 04
2007	NH_3_-N	3.82E + 05	1.43E + 04	3.51E + 04	2.13E + 05
	TN	1.55E + 06	2.11E + 04	1.02E + 05	7.05E + 05
	TP	1.59E + 05	9.24E + 02	5.78E + 03	2.52E + 04
2006	NH_3_-N	4.55E + 05	1.84E + 04	2.50E + 04	2.44E + 05
	TN	1.35E + 06	1.86E + 04	7.14E + 04	7.74E + 05
	TP	1.22E + 05	1.07E + 03	3.22E + 03	3.34E + 04

**Table 3:** Information on the eight rivers draining into the coastal waters of China.

Rivers	Locations	DrainageArea(10^4^ km^2^)	AnnualDischarge(10^8^ m^3^)	Sea Basin	MonitoringSites	Longitude (E) andlatitude (N)
Yangtze	Middle	170.54	8964	East China Sea	Shanghai	121.88°, 31.07°
Huanghe	North	68.22	341.2	Bohai Sea	Dongying	118.32°, 37.54°
Liaohe	Northeast	12.76	12.76	Bohai Sea	Panjin	121.89°, 41.03°
Haihe	North	5.22	15.55	Bohai Sea	Tanggu	117.73°, 38.99°
Huaihe	North Central	13.16	13.16	Yellow Sea	Huaian	118.46°, 33.07°
Qiangtangjiang	Southeast	2.30	198.9	East China Sea	Hangzhou	120.16°, 30.21°
Mingjiang	Southeast	5.85	573.9	East China Sea	Fuzhou	119.57°, 26.14°
Zhujiang	South	41.52	2833	South China Sea	Guangzhou	113.53°, 22.99°
