# Supplementary material for: Nutrient Loads Flowing into Coastal Waters from the Main Rivers of China (2006–2012)
Source: Sci Rep. 2015 Nov 19;5:16678. doi: 10.1038/srep16678 (PMC4652226; doi:10.1038/srep16678)
Supplement: Supplementary Information [file srep16678-s1.doc]

**Nutrient Loads Flowing into Coastal Waters from the Main Rivers of China (2006-2012)**

Yindong Tong^1, 2^, Yue Zhao^3*^, Gengchong Zhen^4^, Jie Chi^1^, Xianhua Liu^1^, Yiren Lu^1^, Xuejun Wang^2, 5^, Ruihua Yao^3^, Junyue Chen^6^, Wei Zhang^4*^

1. School of Environmental Science and Engineering, Tianjin University, Tianjin, 300072, China

2. Ministry of Education Laboratory for Earth Surface Processes, Peking University

3. Chinese Academy for Environmental Planning, Beijing, 100012, China

4. School of Environment and Natural Resources, Renmin University of China, Beijing, 100872, China

5. College of Urban and Environmental Sciences, Peking University, Beijing, 100871, China

6. Yang Ming Institute, Ningbo University, Ningbo, 315122, China

**Water discharges of the rivers**

The total water discharge of the rivers flowing into the Pacific Ocean was about 1.4×10^12^ m^3^ in 2012, with over 3/4 of the water flowing into the East China Sea. Yearly variation in the water discharge was observed. For example, in 2007, the total water discharge of the selected rivers was 1.1×10^12^ m^3^, 22% higher compared to the value in 2011. The river with the largest annual water flow was the Yangtze River, accounting for over 50% of the total water discharge flowing into the ocean. In the Yangtze River, the largest annual water flow occurred in 2010, reaching as high as 1.0×10^12^ m^3^. The water discharges of the rivers in the southern part of China were significantly higher than those in the North, and decreased significantly from south to north. For example, the sum of the annual water flow for the Huanghe River, Liaohe River and Haihe River was only 0.2×10^11^ m^3^ in 2011, while the corresponding value for the Yangtze River was 6.7×10^11^ m^3^. Seasonal variation of the water discharge was observed in all of the selected rivers. Higher flows occurred more often during the rainy season, and over 62% of the total water discharges occurred from April to September. The highest monthly discharge of the rivers always occurred from July to September.

***Due to the lack of data, the TN loads were not included for the Liaohe River.**

**Figure S1. Monthly variations in NH_3_-N, TN and TP loads from 2006 to 2012 (tons)**

**Figure S2. Monthly water discharges of the eight rivers from 2006 to 2012**

**Table S1. Seasonal variations in nutrients loads from the eight rivers during 2006-2012 (10^4^** tons)

|  |  | **Spring** | | | **Summer** | | | **Autumn** | | | **Winter** | | |
| --- | --- | --- | --- | --- | --- | --- | --- | --- | --- | --- | --- | --- | --- |
|  |  | **NH_3_-N** | **TN** | **TP** | **NH_3_-N** | **TN** | **TP** | **NH_3_-N** | **TN** | **TP** | **NH_3_-N** | **TN** | **TP** |
| **Yangtze** | **2006** | 18.07 | 35.56 | 3.70 | 15.49 | 43.31 | 3.58 | 6.95 | 35.65 | 2.56 | 3.23 | 20.61 | 2.38 |
|  | **2007** | 10.28 | 24.80 | 2.76 | 5.77 | 37.10 | 3.53 | 13.85 | 68.33 | 6.92 | 6.73 | 22.83 | 2.38 |
|  | **2008** | 10.59 | 25.79 | 1.49 | 9.37 | 45.15 | 3.98 | 9.99 | 69.56 | 6.00 | 4.82 | 31.56 | 2.85 |
|  | **2009** | 6.29 | 23.73 | 2.55 | 8.94 | 48.46 | 4.19 | 7.85 | 57.35 | 5.29 | 2.48 | 19.85 | 2.79 |
|  | **2010** | 6.36 | 31.77 | 4.04 | 13.92 | 68.45 | 6.29 | 16.15 | 79.24 | 6.23 | 6.37 | 29.96 | 3.68 |
|  | **2011** | 7.67 | 22.86 | 2.95 | 7.57 | 40.24 | 4.46 | 10.24 | 59.16 | 6.04 | 4.33 | 39.24 | 3.27 |
|  | **2012** | 9.59 | 40.78 | 3.61 | 6.60 | 68.13 | 7.13 | 5.70 | 63.07 | 8.74 | 2.53 | 31.82 | 4.28 |
| **Huanghe** | **2006** | 0.16 | 0.20 | 0.01 | 0.52 | 0.51 | 0.04 | 0.39 | 0.67 | 0.02 | 0.12 | 0.23 | 0.01 |
|  | **2007** | 0.06 | 0.11 | 0.00 | 0.21 | 0.27 | 0.01 | 0.41 | 0.94 | 0.03 | 0.26 | 0.49 | 0.02 |
|  | **2008** | 0.12 | 0.20 | 0.01 | 0.25 | 0.46 | 0.02 | 0.20 | 0.43 | 0.01 | 0.10 | 0.27 | 0.01 |
|  | **2009** | 0.07 | 0.13 | 0.00 | 0.14 | 0.33 | 0.02 | 0.24 | 0.42 | 0.03 | 0.26 | 0.33 | 0.01 |
|  | **2010** | 0.12 | 0.15 | 0.01 | 0.23 | — | 0.04 | 0.58 | 0.80 | 0.03 | 0.14 | 0.25 | 0.02 |
|  | **2011** | 0.03 | — |  | 0.10 | 0.22 | 0.01 | 0.46 | 0.58 | 0.03 | 0.42 | 0.75 | 0.04 |
|  | **2012** | 0.11 | 0.54 | 0.01 | 0.26 | — | 0.02 | 0.61 |  | 0.06 | 0.27 |  | 0.03 |
| **Liaohe** | **2006** | 0.11 | — | 0.00 | 0.22 | — | 0.01 | 0.07 |  | 0.00 | 0.01 |  | 0.00 |
|  | **2007** | 0.03 | — | 0.00 | 0.09 | — | 0.01 | 0.08 |  | 0.00 | 0.02 |  | 0.00 |
|  | **2008** | 0.08 | — | 0.00 | 0.18 | — | 0.01 | 0.11 |  | 0.03 | 0.01 |  | 0.01 |
|  | **2009** | 0.05 | — | 0.00 | 0.08 | — | 0.02 | 0.02 |  | 0.01 | 0.01 |  | 0.00 |
|  | **2010** | 0.02 | — | 0.00 | 0.11 | — | 0.01 | 0.52 |  | 0.03 | 0.17 |  | 0.01 |
|  | **2011** | 0.10 | — | 0.00 | 0.07 | — | 0.01 | 0.07 |  | 0.02 | 0.02 |  | 0.01 |
|  | **2012** | 0.02 | — | 0.00 | 0.07 | — | 0.01 | 0.08 |  | 0.01 | 0.04 |  | 0.01 |
| **Huaihe** | **2006** | 0.71 | 1.22 | 0.05 | 0.35 | 1.13 | 0.04 | 1.13 | 3.71 | 0.19 | 0.15 | 0.72 | 0.03 |
|  | **2007** | 0.87 | 1.80 | 0.06 | 0.33 | 0.93 | 0.04 | 2.12 | 6.89 | 0.46 | 0.19 | 0.57 | 0.02 |
|  | **2008** | 0.28 | 0.36 | 0.02 | 0.23 | 0.53 | 0.05 | 0.77 | 1.62 | 0.16 | 0.20 | 0.38 | 0.03 |
|  | **2009** | 0.09 | 0.14 | 0.01 | 0.15 | 0.32 | 0.02 | 0.52 | 0.80 | 0.11 | 0.17 | 0.28 | 0.03 |
|  | **2010** | 0.36 | 0.47 | 0.04 | 0.31 | 0.59 | 0.06 | 1.07 | 1.76 | 0.20 | 0.15 | 0.30 | 0.03 |
|  | **2011** | 0.09 | 0.12 | 0.01 | 0.06 | 0.09 | 0.01 | 0.25 | 0.42 | 0.04 | 0.22 | 0.30 | 0.03 |
|  | **2012** | 0.14 | 0.18 | 0.01 | 0.08 | 0.13 | 0.01 | 0.39 | 0.60 | 0.06 | 0.12 | 0.16 | 0.01 |
| **Minjiang** | **2006** | 0.28 |  |  | 0.95 |  |  | 0.48 |  |  | 0.12 |  |  |
|  | **2007** | 0.28 |  | 0.06 | 0.33 |  | 0.06 | 0.34 |  | 0.06 | 0.02 |  | 0.00 |
|  | **2008** | 0.20 |  | 0.04 | 0.55 | 2.49 | 0.10 | 0.39 | 1.90 | 0.04 | 0.18 | 0.90 | 0.02 |
|  | **2009** | 0.22 | 1.06 | 0.03 | 0.40 | 1.94 | 0.07 | 0.38 | 1.86 | 0.08 | 0.17 | 0.97 | 0.02 |
|  | **2010** | 0.36 | 2.08 | 0.05 | 1.39 | 7.93 | 0.34 | 0.47 | 2.57 | 0.09 | 0.15 | 0.83 | 0.04 |
|  | **2011** | 0.18 | 0.99 | 0.03 | 0.33 | 1.89 | 0.05 | 0.16 | 1.14 | 0.04 | 0.10 | 0.92 | 0.03 |
|  | **2012** | 0.35 | 2.79 | 0.12 | 0.60 | 5.09 | 0.20 | 0.24 | 2.08 | 0.08 | 0.44 | 2.36 | 0.09 |
| **Zhujiang** | **2006** | 3.01 | 10.03 | 0.43 | 9.60 | 24.43 | 1.04 | 8.84 | 34.58 | 1.61 | 2.95 | 8.37 | 0.26 |
|  | **2007** | 2.73 | 6.69 | 0.50 | 10.85 | 18.87 | 0.81 | 7.24 | 35.29 | 1.00 | 0.53 | 9.68 | 0.21 |
|  | **2008** | 5.59 | 11.04 | 0.35 | 14.54 | 35.05 | 1.64 | 5.71 | 36.72 | 1.62 | 3.07 | 18.95 | 0.66 |
|  | **2009** | 3.37 | 13.56 | 0.34 | 9.58 | 26.49 | 0.57 | 4.53 | 26.85 | 0.84 | 2.50 | 8.26 | 0.25 |
|  | **2010** | 3.62 | 11.48 | 0.34 | 16.07 | 43.21 | 2.07 | 6.18 | 26.71 | 0.95 | 2.93 | 13.75 | 0.30 |
|  | **2011** | 3.02 | 8.81 | 0.34 | 6.56 | 18.93 | 0.94 | 3.04 | 10.06 | 0.48 | 1.28 | 11.80 | 0.41 |
|  | **2012** | 3.56 | 11.20 | 0.35 | 11.39 | 37.34 | 1.53 | 4.28 | 20.79 | 1.13 | 2.69 | 10.83 | 0.46 |
| **Qiantangjiang** | **2006** |  |  |  |  |  |  |  |  |  |  |  |  |
|  | **2007** | 0.17 |  | 0.04 | 0.20 |  | 0.03 | 0.07 | 0.58 | 0.01 | 0.06 |  |  |
|  | **2008** | 0.10 | 0.63 | 0.02 | 0.68 | 2.27 | 0.07 | 0.06 | 0.56 | 0.02 | 0.06 | 0.42 | 0.02 |
|  | **2009** | 0.12 | 0.78 | 0.02 | 0.12 | 0.91 | 0.03 | 0.14 | 0.92 | 0.03 | 0.10 | 0.56 | 0.02 |
|  | **2010** | 0.35 | 2.30 | 0.10 | 0.44 | 2.28 | 0.10 | 0.18 | 1.17 | 0.05 | 0.06 | 0.84 | 0.02 |
|  | **2011** | 0.10 | 0.71 | 0.02 | 0.17 | 2.68 | 0.07 | 0.06 | 0.43 | 0.02 | 0.07 | 0.77 | 0.02 |
|  | **2012** | 0.37 | 2.43 | 0.07 | 0.40 | 2.76 | 0.11 | 0.06 | 1.10 | 0.04 | 0.13 | 1.23 | 0.03 |
| **Haihe** | **2006** | 0.03 | 0.06 | 0.00 | 0.06 | 0.09 | 0.00 | 0.06 | 0.07 | 0.00 | 0.05 | 0.03 | 0.00 |
|  | **2007** | 0.05 | 0.06 | 0.00 | 0.05 | 0.06 | 0.00 | 0.08 | 0.10 | 0.00 | 0.08 | 0.08 | 0.00 |
|  | **2008** | 0.04 | 0.06 | 0.00 | 0.08 | 0.09 | 0.00 | 0.19 | 0.33 | 0.01 | 0.06 | 0.08 | 0.00 |
|  | **2009** | 0.09 | 0.12 | 0.00 | 0.26 | 0.30 | 0.01 | 0.13 |  | 0.01 | 0.19 | 0.14 | 0.01 |
|  | **2010** | 0.07 |  | 0.00 | 0.07 | 0.03 | 0.00 | 0.09 | 0.11 | 0.01 | 0.06 | 0.06 | 0.01 |
|  | **2011** | 0.02 |  | 0.00 | 0.01 |  | 0.00 | 0.07 |  | 0.01 | 0.01 |  | 0.01 |
|  | **2012** | 0.02 | 0.03 | 0.00 | 0.03 | 0.04 | 0.00 | 0.14 | 0.28 | 0.02 | 0.02 | 0.07 | 0.01 |
| *For the season with data missing for all the three months, the seasonal flux was not included.  **For the Liaohe River, the fluxes of TN was not included | | | | | | | | | | | | | |
